# I Hear You Eat and Speak: Automatic Recognition of Eating Condition and Food Type, Use-Cases, and Impact on ASR Performance

**DOI:** 10.1371/journal.pone.0154486

**Published:** 2016-05-13

**Authors:** Simone Hantke, Felix Weninger, Richard Kurle, Fabien Ringeval, Anton Batliner, Amr El-Desoky Mousa, Björn Schuller

**Affiliations:** 1 Machine Intelligence & Signal Processing Group, MMK, Technische Universität München, München, Germany; 2 Chair of Complex & Intelligent Systems, University of Passau, Passau, Germany; 3 Pattern Recognition Lab, Friedrich-Alexander-Universität Erlangen-Nürnberg, Erlangen, Germany; 4 Department of Computing, Imperial College London, London, United Kingdom; University of Kent, UNITED KINGDOM

## Abstract

We propose a new recognition task in the area of computational paralinguistics: automatic recognition of eating conditions in speech, i. e., whether people are eating while speaking, and what they are eating. To this end, we introduce the audio-visual iHEARu-EAT database featuring 1.6 k utterances of 30 subjects (mean age: 26.1 years, standard deviation: 2.66 years, gender balanced, German speakers), six types of food (Apple, Nectarine, Banana, Haribo Smurfs, Biscuit, and Crisps), and read as well as spontaneous speech, which is made publicly available for research purposes. We start with demonstrating that for automatic speech recognition (ASR), it pays off to know whether speakers are eating or not. We also propose automatic classification both by brute-forcing of low-level acoustic features as well as higher-level features related to intelligibility, obtained from an Automatic Speech Recogniser. Prediction of the eating condition was performed with a Support Vector Machine (SVM) classifier employed in a leave-one-speaker-out evaluation framework. Results show that the binary prediction of eating condition (i. e., eating or not eating) can be easily solved independently of the speaking condition; the obtained average recalls are all above 90%. Low-level acoustic features provide the best performance on spontaneous speech, which reaches up to 62.3% average recall for multi-way classification of the eating condition, i. e., discriminating the six types of food, as well as not eating. The early fusion of features related to intelligibility with the brute-forced acoustic feature set improves the performance on read speech, reaching a 66.4% average recall for the multi-way classification task. Analysing features and classifier errors leads to a suitable ordinal scale for eating conditions, on which automatic regression can be performed with up to 56.2% determination coefficient.

## Introduction

*Talking whilst eating is like talking whilst not eating: good talk is good and bad talk is bad*.

Shaykh al-Albaani

When dealing with speech, we normally either want to recognise *what* has been spoken (Automatic Speech Recognition, ASR), or *how* it has been spoken (Computational Paralinguistics, CP [1]). Much of ASR research has been focussing on the ‘typical’ speaker, i. e., a speaker which is representative for the speech community and at the same time, does not display any non-typical states such as emotions or stress, or traits such as pathology, strong idiosyncratic traits, and suchlike.

In contrast, CP aims at identifying exactly these states and traits, mainly to endow future technical systems with the ability to interpret and react to them appropriately. Identifying characteristic features of atypical speech is also expected to help ASR performance, since dedicated techniques, e. g., for model adaptation, could be employed.

Our research is embedded in the European iHEARu project [2], which pursues both ‘universal’ and ‘holistic’ analysis of speech, i. e., analysing multiple attributes simultaneously and jointly. Many speech traits are still not covered by existing CP research, and we lack both labelled data and insights into relevant acoustic features. In this article, we target eating condition—i. e., recognising if people are eating while talking, and if yes, what they are eating –, and we identify discriminative acoustic features and investigate adequate modelling of this task. To the best of our knowledge, neither this task nor ASR for ‘speech under eating’ have been attempted in the literature. At second thought, this comes as a surprise; after all, eating is one of the basic activities of human beings, and speaking while eating is possible and encountered quite often, albeit it is often more or less stigmatised as being unpolite or even more or less disgusting, according to the culture.

**Related Work:** A few studies do address ASR under various speaking conditions, for instance, stress [3], emotion [4], or non-native accent [5]. Conversely, automatic assessment of the speaking condition itself (CP) has been addressed in several studies, for example, non-native speech [6, 7], pathological speech [8–11], or sleepiness as short- or medium-term trait [12].

Furthermore, there are a few studies investigating speaking whilst speakers bite on a block [13]; muscle movements under speaking and eating are dealt with in [14]. Regarding phonological approaches towards speech while eating, we only are aware of one study that was not intended to be taken fully seriously [15]. For subjects chewing gum, [16] showed “a great deal of individual variation in articulation and acoustics between speakers, but consistent productions and maintenance of relative acoustic distances within speakers.”

Another body of literature worth to mention concerns the study of the sounds generated by biting and chewing different types of food. The spectral composition of eating sounds generated by different types of food, e.g., crispy, crunchy, and crackly, was analysed in [17], where the fundamental frequency of those sounds has been found to be a good indicator of the type of food. Subjective evaluations on the pleasantness of food sounds produced by biting and chewing various items of food have been carried out in [18]; crispy and crunchy foods appeared to be the most appealing foods according to the generated sounds. Sensory qualities of food sounds were evaluated in [19], using 15 sensory acoustical quality attributes. Data were analysed with the non-metric dimensional scaling (NMDS) algorithm from symmetric dissimilarity matrices. Results suggested two sensory criteria for distinguishing food sounds: the continuousness or evenness of the sound, and the loudness. Finally, subjective tests were performed in [20]; participants had to recognise the type of food according to the sound generated when crushing different types of food items. The author reported that the performance obtained by human judges does not seem to depend on the familiarity or the class (e.g., vegetable, cracker) of food.

**Motivation:** Independent of this white spot in the literature—the automatic recognition of eating while speaking, the list of applications for a system able to detect speech under eating condition is rich. In the following, we will give some possible applications.

- **Automatic Speech Recognition**: When interacting with systems able to recognise speech in our daily live routine, all sorts of activities influence the quality of our speech. For example, in [21] it is reported that medical doctors using dictation software for their medical reports dictate while eating and drinking besides other disturbance factors such as cell phone, car or noise-rich acoustics, or exercising on treadmills. In general, one can expect degradation to the speech recognition accuracy.
  However, adapting acoustic (and linguistic) models is known to help in other cases such as speech affected by emotion [22]. Thus, a system that knows that speech is uttered while eating, and that knows even the type of food, can adapt to cover such degradation. In addition, confidence measures can be adapted if the system is aware of a likely degradation due to eating—again, this can be fit to the type of food.
- **Speaker Identification and Verification**: Similar to the above, degradation can also be expected for the recognition or identification of a speaker. For example, in [23] it was shown that alcohol intoxication of speakers has a negative influence on speaker recognition. Again, either one could train suited models (e. g., asking for eating during enrollment of the speaker) for adaptation or—probably more realistic in most use-cases—simply identify that the speech is under eating condition and ask a speaker to speak with empty mouth or adapt confidence measures in access systems.
- **Computational Paralinguistics**: One step further, other paralinguistic analysis tasks will benefit from the knowledge of eating condition when analysing speech, e. g., when in search of cues of emotion, personality or other speaker states and traits. For example, in [2] it could be shown that commonly training states and traits of speakers helps to improve the recognition of each individual one. Other options include adaptation of paralinguistic models or adaptation of confidence measures if eating condition is detected. Interestingly, the knowledge of eating condition can also directly serve as feature for some paralinguistic analysis tasks such as estimation of the stress-level, personality or social class in the sense of ‘this person also speaks while eating’.
- **Health monitoring**: There is quite some interest in monitoring automatically eating and drinking patterns—also termed as Monitoring of Ingestive Behaviour (MIB), e. g., by video signals [24], accelerometers [25], piezoelectric strain gauge sensors capable of detecting skin motion in the lower trachea [26] or movement of the lower jaw [27] or also by acoustic sounds of chewing [28, 29], or swallowing [30–32], where also ‘contamination’ by speech is considered among other disturbances such as motion. The motivation for MIB includes observation of abnormal eating patterns and prevention of obesity or overweight and eating disorders. One could further think of automatic reminders after medical treatment of the teeth that requires a time period without food intake such as after visiting the dentist or for tooth protection.
  Smart monitoring of dietary programs can also include analysis of the type of food intake, e. g., by ‘hearing’ if you really eat that apple or rather biscuits. Food that breaks into smaller pieces such as crisps or biscuits can make elderly people and especially Parkinson’s patients choke and even endanger their life [33, 34]. Especially in the case of Parkinson’s disease dementia, it can thus be advisable to automatically monitor the type of food consumed by such patients [35].
- **Life-logging**: Measuring oneself automatically for all kinds of purposes has become a recent trend—this includes acoustic monitoring [36] of activities: by a specially crafted wearable acoustic sensor, the authors in [36] were able to recognise sounds produced in the throat area for the recognition of eating, drinking, speaking, laughing, and coughing. Such data can be uploaded into social networks or simply kept for oneself for automatic diaries. Under realistic conditions, the authors report 71.5% recognition rate for these classes excluding coughing—a ‘speech under eating’ class is, however, not modelled. A special custom-built piezoelectric microphone is used for similar classes’ recognition in [37]. Further, an early work [38] reports slightly more optimistic results of “up to 99% accuracy” for acoustic eating recognition and between “80% to 100% on food type classification”.
- **Social Competence**: In future intelligent systems and robots, social skills will play an increasingly important factor. In terms of our application, knowledge of speech while eating will allow such systems to lead a dialogue more ‘human-like’ or—if desired—politely, such as not disturbing or pushing for a response when detecting speech while eating, or simply wishing a ‘bon appétit’.
- **Advertisement Placement**: One can think also of commercial interest in presenting food advertisement—even such that fits the current intake of a user, e. g., when controlling the TV, game console or similar by speech under eating.
- **Smart Assistance**: In ‘smart’ environments such as smart homes, smart cities, smart factories, or smart cars, it is often desirable to monitor the activity of the users, e. g., to predict likely next desires or needs of the users. Previous work in this field includes activity recognition by sound in speech controlled smart homes [39]. Use-cases could include telephone assistance if the user is eating (excusing the user for some time or—depending on the call-type such as an official call—waiting until eating stops or warning the user before accepting the call), for example in the car. Care-taking robots could support healthier live styles by offering fruits when hearing chips and cookie crunches. Finally, smart-cameras could wait if self-portraits are shot by voice activation if eating condition is recognised.
- **Security Monitoring**: In high sanitation and ultra-clean environments such as chip or pharmaceutical production, speech controlled systems could be alarmed when the user is apparently consuming food in the workspace. Further, spoken-interaction-based virtual companions in vehicles could expect reduced attention during eating or fatigue after eating of their conductors.
- **Behavioural Tutoring**: A number of Serious Digital Games target the training and tutoring for social behaviour such as in the MASELTOV project for immigrants or in the TARDIS project for young adults’ first job interview [40]. Such a program can also detect speech under eating condition and give feedback on its appropriateness.
- **Robust Multimodality**: In today’s increasingly multimodal human-machine interaction, knowledge of speech under eating condition can be important for processing other modalities such as facial action unit recognition and prediction or other bio-sensors [41]. In fact, also for facial expression analysis even information on the type of food being taken in will be of interest.
- **Transcription**: In automatic transcription of movies or broadcasts, etc., for a deaf audience, it may be of interest to transcribe also information on speech under eating condition to make this detail available to them. In some delicate scenes such as a servant deliberately speaking to a superior while eating, this may be very relevant.
- **Forensics**: In speech forensics, knowledge of food consumption can be a relevant detail for investigations based, e. g., on a call.
- **Ethnography of Communication**: Eating and speaking are definitely amongst the most important activities for human kind, both for surviving and cultural exchange in societies. The ethnography of communication [42] (originally called ‘ethnography of speaking’ [43]) analyses the components of communicative events within a culture. As [44] puts it: “Speaking and Eating are essential communicative systems. Together they constitute the nature and structure of meals. … the ethnography of communication can document actual speech and dialogue patterns in different types of meals and eating occasions of the present.” The seminal work of [45] documented the changes of allowed and stigmatised behaviour at the table in different cultures and different times; still nowadays, smacking, slurping, or spitting at the table and/or in public are stigmatised in some cultures while being allowed in other ones. Apart from detecting less accepted eating manners, a tool monitoring eating conditions could as well be used for studying sequences of speech, eating, and speech under eating, together with other non-verbal behaviour, in formal and less formal settings, both within our own much studied and less studied cultures and languages.
- **Speech Enhancement**: Last but definitely not least, one can think also of filtering out different kind of food noises in speech in real time to allow eating while speaking. Such an application can detect speech under eating condition and give clean speech to the conversation partners which could be applied for possible situations like having a long business telephone conference or working as an employee in a call-center.

Speaking while eating is a new topic in the field of Computational Paralinguistics that will definitely attract researchers. Such interest from the scientific community has already been observed at the latest edition of the Interspeech Computational Paralinguistics Challenge (ComParE 2015) [46], where the automatic recognition of the eating condition was featured as a task. The outcome of this sub-challenge will be detailed at the end of the paper.

Motivated accordingly, let us next describe the recording of the first speech under eating condition database before describing some first experimental results on the data and drawing some conclusions.

## Database

For our recordings the subjects were invited into a room of the Technische Universität München (Munich University of Technology). To achieve a high audio and video quality, the recordings took place in a comparably low reverberant office room with equal set-up and room conditions for each recording session. To guarantee equal light conditions, the roll-down curtains were kept closed during all recordings. Instead, a light source in top and front of the subjects was used, and a portable floodlight was directed to the white ceiling and illuminated the subjects by generating a weak large-area reflection.

Before the recordings, the participants read through an information sheet telling them what they were required to do in the experiment, and describing each task. Then, they had to sit down in front of the recording equipment. Behind them we set up a white screen, which provided a constant white background for each recording. The subjects were instructed on the procedures by an experimental supervisor. For the recordings, we implemented a program with a graphical user interface (GUI) in Matlab, enabling the subjects to indicate the start and end points of their utterances by themselves, in order to facilitate the segmentation of the recordings.

Prior to the actual recording, subjects performed practice trials to familiarise themselves with the procedure. The experimental supervisor was attendant at all times for questions, help, and to serve the food, but was sitting behind the white screen to ensure that the subjects felt unobserved while they were eating and speaking.

For our video recordings, we used a Logitech HD Pro Webcam C920 placed on top of the computer monitor on the desk in front of the subjects. The video files are coded with an MJPEG codec with a frame rate of 30 fps and a resolution of 1280 x 720 pixels. The audio-stream was taken with an AKG HC 577 L headset microphone with a sampling frequency of 44.1 kHz in mono with 24 bits per sample. The external sound card MAudio Fast Track C400 was used to ensure high quality in the digitisation of the speech signal. These technical information are summed up in Table 1.

**Table 1:** Technical setup for the recording of the audio-visual streams.

**Audio**	
Tools	AKG HC 577 L headset
	MAudio Fast Track C400
Sample rate	44.1 kHz mono
Bit depth	24 bits per sample
**Video**	
Tool	Logitech HD Pro Webcam C920
Codec	MJPEG
Framerate	30 fps
Resolution	1280 x 720 pixels

In order to achieve maximum synchronisation of our recorded audio and video streams, we used a trigger implemented in Matlab, and created timestamps every time a subject pressed the start and stop buttons in the recording GUI. This allowed us afterwards to synchronise audio and video data properly.

The participant consent was documented by a written consent statement. The consent forms have been checked and approved by an ethic committee (TUM IRB). The participants of the recordings were recruited via ‘word-of-mouth recommendation’. The experiment involved 30 subjects, which gave their written permission to data recording, storage, and distribution for scientific purposes. Further, the subjects gave information about some personal data and health issues like speech impediment, vocal tract dysfunction, difficulties in swallowing, heart and circulatory problems, toothaches, being vegetarian, smoking, and alcohol consumption, which can be found in Table 2. The data are stored in an anonymous form, so no identifying information was collected from participants at all. Further, the subjects gave us their written consent to illustrate audio and/or video examples as well as figures in scientific publications.

**Table 2:** Number of subjects having special health issues.

Health issue	Never	Rarely	Sometimes	Regularly	Often
Speech impediment	26	2^1^	2^1^	0	0
Vocal tract dysfunction	29	0	1^1^	0	0
Difficulties in swallowing	28	2^1^	0	0	0
Heart and circulatory problems	28	2	0	0	0
Toothaches	27	2	1	0	0
Being vegetarian	28	2^2^	0	2^3^	0
Smoking	13	2	8	4	3
Alcohol consumption	1	5	14	3	7

^1^ The subjects have masticatory disturbance and feel difficulty in chewing and swallowing under illness. Under normal health circumstances—as during the recordings—no subject had any speech impediment, vocal tract dysfunction or masticatory disturbance.

^2^ The subjects eat fish but no meat.

^3^ The subjects prefer vegetarian food whenever possible.

They optionally filled in further information like their age, height, weight, nationality, and their German proficiency. Out of those 30 speakers, 15 subjects are female and 15 male, with a mean age of 26.1 years and a standard deviation of 2.7 years. 27 of the subjects are German native speakers; one is Chinese, one is Indian, and one has a Tunisian origin, all having a close-to-native proficiency in German.

We decided to have food classes with partly similar consistency (e.g., Crisps and Biscuits) and partly dissimilar consistency (e.g., Nectarine vs. Crisps), thus providing a fair variety of speech disturbances while eating. Moreover, the food classes represent snacks which are easy to prepare and hence likely to be encountered in practical scenarios, have a low probability of allergies, and enable the subjects to speak while eating. We always choose the approximately same medium-ripe fruit, which was ready to eat and not too unripe and sour. All fruits were cut and served directly before the recordings.

In order to control for the amount of food being consumed, and in particular to encourage subjects to actually eat while speaking, an experimental supervisor provided the subjects with a serving of fixed size prior to the recording of each utterance. Each recorded utterance includes speech simultaneous with eating. The read sentences had a recording length which made it possible for the subject to record them without having to stop eating. For the spontaneous narrative utterances, subjects just stopped talking, for example, to put the food to the other side of the mouth, otherwise they were eating and chewing while speaking. Naturally, since the mouth cavity is generally larger for men than for women and differs from subject to subject, the actual amount of food consumed varies a bit among subjects. The serving size was personalised and chosen such as to enable a significant effect on the subjects’ speech, while making the production of speech feasible and audible. The chosen food, together with an approximate amount of food served per utterance, is shown in Table 3. The subjects were advised not to eat food during the experiment they are allergic to or they did not like to eat for any other personal reason.

**Table 3:** Chosen food classes and amount of food served to the subjects while recording each utterance.

Food	ID	Weight [g]
Apple	Ap	11–15
Nectarine	Ne	17–20
Banana	Ba	22–26
Haribo Smurfs ^1^	Ha	5
Biscuit	Bi	5–6
Crisps	Cr	3–4

^1^Haribo Smurf is a specific type of jelly gum.

Subjects were asked to self-report on how much they like each sort of food they were going to eat during the experiment. This was achieved by setting a continuous slider to a value ranging between 0–*dislike extremely* and 1–*like extremely*. After the recording session, the subjects were asked to specify on a 5-point Likert scale the difficulties they encountered in eating each sort of food while speaking. Statistics from the self-reports show that Banana was perceived as the most liked type of food and the less difficult to eat, whereas Biscuit was the most disliked and difficult type of food to eat, cf. Table 4.

**Table 4:** Self-reporting on likability and difficulty of eating of food classes rated by all subjects.

Food	Likability	Difficulty
Apple	.73 (.20)	.63 (.24)
Nectarine	.76 (.21)	.47 (.23)
Banana	.77 (.20)	.43 (.25)
Haribo Smurfs	.67 (.29)	.45 (.26)
Biscuit	.56 (.25)	.67 (.26)
Crisps	.68 (.28)	.54 (.26)

The ratings of likability are in a [0–1] scale (dislike extremely: 0 and like extremely: 1). The ratings of difficulty of eating are converted from a 5-point Likert into a [0–1] scale (very easy: 0 and very difficult: 1), to ease comparison with reports on likability; [mean value] (standard deviation).

The recorded data consists of read and spontaneous speech. For read speech, the German version of the frequently used standard text in phonetics “The North Wind and the Sun” (“Der Nordwind und die Sonne” in German) was chosen [47]. The text is phonetically balanced and contains 108 words (71 distinct) with 172 syllables. The subjects had to read the whole text with each sort of food. Spontaneous speech was recorded by giving the subjects different topics to talk about, e. g., their favourite travel destination, genre of music, or sports activity. Speech of only one topic was recorded per consumed piece of food. A typical session of one subject lasted about one hour. All in all, for the 30 subjects, 1.6 k utterances and 3:20 hours of speech were recorded, cf. Table 5.

**Table 5:** Statistics of the iHEARu-EAT database.

	Read		Spontaneous	
Class	#	Duration	#	Duration
Apple	196	24:47	28	4:00
Nectarine	196	25:00	28	3:37
Banana	210	25:21	30	3:41
Haribo Smurfs	189	22:57	27	3:38
Biscuit	203	25:47	29	4:08
Crisps	210	25:58	30	3:44
No Food	210	23:03	30	4:13
Total	1414	2:53:01	202	27:05

Number (#) and duration of speech utterances per class for read and spontaneous conditions. The slight difference in the number of utterances per class is due to the fact that some subjects chose not to eat all types of food.

Even though the recording procedure allowed an automatic segmentation of speech data, we manually segmented the obtained utterances, in order to remove parts (i.e., beginning or ending of a sentence) that only contained ‘eating noise’. This additional segmentation procedure guarantees that our analysis is solely based on speech data under eating condition, and not on a mixture of speech with additional eating / biting / chewing sounds.

**Fig 1:**
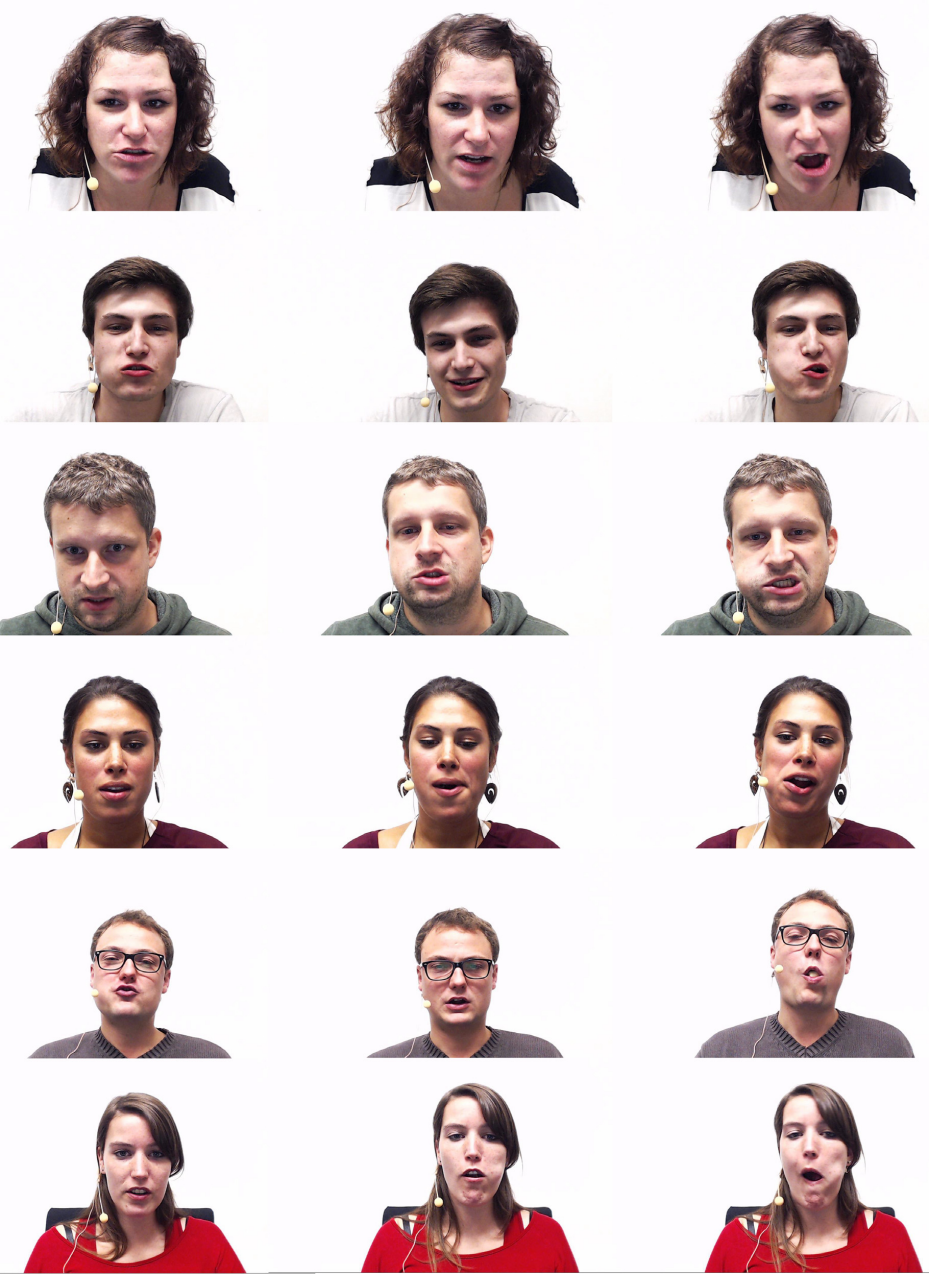
Exemplary subjects of the iHEARu-EAT database while recording an utterance without eating food (left), eating a banana (middle) and eating crisps (right). Unusual configurations of the supra-glottal part of the vocal tract are clearly visible for the eating conditions.

A selection of subjects while recording an utterance—first without eating food (left), then eating a banana (middle) and finally eating crisps (right)—is shown in Fig 1. One can easily see that the condition of speaking while eating imposes unusual configurations in the supra-glottal part of the vocal tract, which thus introduces important disturbances in the production of phonemes, but also on their co-articulation.

The database, including the audio-visual data and transcriptions, is licensed under the Creative Commons BY-NC-SA terms and is publicly available for academic research purpose (http://openaudio.eu).

## Automatic Speech Recognition Experiments

We perform Automatic Speech Recognition (ASR) for the iHEARu-EAT dataset using an acoustic Hidden Markov Model with Gaussian Mixture Densities (HMM-GMM) system trained using the Kaldi toolkit [48]. The acoustic front end consists of 13 mel-frequency cepstral coefficients (MFCCs) derived from a bank of 23 filters and augmented with first and second derivatives. The MFCCs are normalised using cepstral mean and variance normalization (CMVN). A linear discriminant analysis (LDA) matrix is used to project the concatenation of seven consecutive feature vectors in a sliding window into 40 dimensions followed by a maximum likelihood linear transformation (MLLT).

The acoustic models are triphone models based on maximum likelihood (ML) training. Speaker variations are compensated by applying speaker adaptive training based on feature space maximum likelihood linear regression (fMLLR). Triphone parameters are tied using an automatically generated classification and regression tree (CART).

The pronunciation model is trained on a lexicon of one million German words using the Phonetisaurus toolkit [49]. The model achieves a word error rate of 9% on a held-out test set of unseen words. This grapheme-to-phoneme conversion model is used to generate pronunciations for words outside our base lexicon.

The initial acoustic model is trained on around 160 hours of transcribed audio material taken from broadcast news domain. The language model (LM) is trained on text corpora that consist of around 307 million running words including data from the newspaper TAZ, and web collected German news articles. The vocabulary is selected out of the text corpora by choosing the 200k top most frequent words in addition to around 6.5k words that occur in the transcription of the acoustic data. Recognition lattices are generated using a small pruned trigram LM, then lattices are rescored with a big non-pruned 4-gram LM. The LMs are smoothed using modified Kneser-Ney (MKN) smoothing [50]. The SRILM toolkit [51] is used for training the LMs.

For training and testing on the iHEARu-EAT dataset, we first make a dataset division such that the training and testing partitions have different speakers. Therefore, for each of the six food types and the No Food data, we selected 26 out of 30 speakers to be used for training and the other 4 to be used for testing. Thus, the system is never trained and tested on the same speakers. The initially trained GMM models are used to produce an alignment, then the whole training data is used to re-train the GMM models including the six types of foods and the No Food data. Then, each type of food is used separately to train an acoustic model.

Table 6 displays the word error rates (WERs) of the ASR task, showing the impact of training with speech data while eating and without eating on the 7-class recognition task. Shown are the WERs as measured on the testing data for each food type as well as for No Food. It can be seen that, in most cases, training on a single type of food performs better in matched testing conditions than training on other unmatched types of food. Note that this is just not the case for Haribo Smurfs and Nectarine. Training on the union of No Food data and All Food data (multi-condition training) leads to the best results for all types of food, but not for the No Food speech. The most probable reason behind that is the increased amount of training data in the case of multi-condition training. Thus, our conclusion is that it is better to train on clear (No Food) speech, for recognizing clear speech. Adding noisy (Food) speech could harm in this case. On the other hand, to recognize noisy speech, the best way is to train on all the available data whether noisy (Food) or clear (No Food), so all conditions are represented in the training set and can be learned accordingly. In this case, there is most probably not much influence of a particular type of food as the effect on the articulation is almost similar in all food types. Therefore, the training could benefit from all data in the multi-condition training to efficiently recognize speech data from a single condition.

**Table 6:** ASR WERs [%] using 7-way acoustic model training on the iHEARu-EAT dataset.

**Train/Test**	**Ap**	**Ne**	**Ba**	**Ha**	**Bi**	**Cr**	**No**
**Ap**	**13.99**	**21.79**	24.54	**30.50**	31.88	28.44	6.42
**Ne**	21.79	24.31	25.46	35.78	34.63	34.40	9.17
**Ba**	17.66	23.17	**24.54**	33.94	34.17	28.44	8.26
**Ha**	20.41	26.38	27.75	32.11	31.88	30.28	11.01
**Bi**	20.18	25.46	32.80	30.96	**27.75**	25.46	11.01
**Cr**	18.81	25.23	26.83	31.65	28.21	**23.39**	8.72
**No**	34.17	47.02	52.29	55.05	61.93	54.36	**3.90**
**No + All**	10.32	16.74	17.20	21.79	16.51	13.99	5.28

## Automatic Classification Experiments

In the following, we show experimental results with the task of classifying the eating condition from audio. Both binary classification (Food / No Food) and 7-way classification (6 food classes or No Food) are investigated. The evaluation procedure consists of a leave-one-speaker-out cross validation (LOSO-CV), which ensures speaker independence in the performance evaluation, as required by many practical applications. Performance is measured as the unweighted average recall (UAR) of the classes, which represents the accuracy in a dataset with equal class priors. This is especially important for the binary task where the class distribution is imbalanced and high accuracy could be achieved by picking the majority class (Food). UAR is calculated by the sum of recall-values (class-wise accuracy) for all classes divided by the number of classes. The great advantage of UAR lies in the fact that, given the number of classes, chance level is always the same, irrespective of the number of cases within classes: For two classes, it is 0.5, for three 0.33, for four 0.25, and so on. By that, UAR meets the requirements for common language measures [52] such as standardisation, ease of interpretation, and plausibility. Together with a confusion matrix and number of cases per class, all information can be extracted. This is a standard measure for evaluating performance in CP and was used as competition measure in the challenge (cf. [1, 53]). For binary classification, it indicates how well both classes are recognised at the standard operating point (class posterior probability = 0.5).

### Classification with ASR-related features

As a first approach, we investigate features derived from ASR output for the above mentioned classification tasks. The hypothesis is that speech under eating condition is harder to process for an ASR system than speech without eating; this would be reflected in objective measurements on the ASR output, such as error rates. Moreover, various consistencies of food may induce different types of deviation in the supra-glottal configuration of the vocal apparatus, and thus in the produced speech waveform. We derived a fairly straightforward set of ASR-related features for our study: Firstly, we use word error rate (WER), as was already proposed in [47] for automatic intelligibility rating—a task that is arguably related to the task at hand. In addition to WER, we also consider character error rate (CER), which is insensitive to errors related to splitting of German compound words (e. g., *Nordwind* vs. *Nord Wind*). Since WER and CER require the reference transcription, which might not be given in practical applications requiring text-independence, we also consider reference-free measurements. The most straightforward one is the log likelihood (LL) log *p*(*w*|**x***_t_*) of the 1-best hypothesis *w* given the acoustic features **x***_t_*, *t* = 1,…,*T*, normalised by the number *T* of the short-term signal frames in an utterance. Furthermore, we assume that a standard beam-search decoder will pursue more potential hypotheses in case of low confidence, so that the real-time factor (RTF), i. e., decoding time over utterance length, would increase in case of low intelligibility.

For ASR, the German LVCSR system described in [54] is used. Triphone acoustic Hidden Markov Models with Gaussian mixture densities (HMM-GMMs), using 39 Perceptual Linear Prediction (PLP) coefficients (including deltas and energy) as acoustic features **x**_*t*_, were trained on 146 hours of German broadcast news speech; the language model was trained on 189 million words of German newspaper texts. We use an ‘out-of-the-box’ system, since here, we are not interested in optimising ASR performance, but using ASR-related measures as features for paralinguistic classification. In particular, no adaptation is performed since that would reduce performance differences between eating and not-eating conditions.

Before turning to automatic classification, we first confirm the usefulness of these features by statistical analysis. Since this is an exploratory study, we use a level of *α* of 0.05 throughout. First, we ran two-sided Welch two-sample t-tests with the binary class labels, e. g., No Food and Food. For CER and WER, we obtained t-statistics of 38.9 and 27.0, respectively, corresponding to a p-value *p* < .001. The sign of the statistics indicates that indeed, the error rate is significantly higher for the Food group. In the same vein, the t-statistic for LL has a negative sign (-21.5), also at *p* < .001. Finally, the RTF exhibits a t-statistic of 44.9 (*p* < .001). Further, for all of the features we find a significantly different distribution among the six food types, providing evidence that they could also be useful for the 7-way classification task. This was verified using an analysis of variance (*p* < .001 for CER, WER and RTF, *p* < .01 for LL).

Starting from these promising results, we investigate the performance of these individual features, as well as their combination, for the binary classification task, i. e., eating vs. not-eating, and the 7-way classification task, i. e., classifying all sorts of food plus not-eating condition. As classifier, we used a SVM, because it has provided high performance in various CP tasks [53, 55]. SVMs are trained using the SMO algorithm implemented in Weka’s SMO class; we used a linear kernel and a complexity of 0.1, all others parameters are set to default.

### Classification with low-level acoustic features

The second approach investigated here is the brute-forcing of low-level acoustic features, which has been successfully applied to a variety of CP tasks [53]. In particular, the feature set designed as baseline for the Interspeech 2013 ComParE challenge [55] and being used for the Interspeech 2015 ComParE challenge [46] is used here, which is extracted using our own open-source toolkit openSMILE [56].

The ComParE feature set contains 6 373 static features—functionals of low-level descriptor (LLD) contours. The LLDs and functionals included in the set are summarised in Tables 7 and 8, respectively. This feature set is the result of continuous refinement of CP-related audio features [53, 55]—the basic idea is to extract frame-wise descriptors such as auditory spectrum, Mel-frequency cepstrum coefficients (MFCCs), loudness, fundamental frequency, and voice quality as well as their delta coefficients, and summarise these contours over the duration of an utterance by providing statistics such as means, moments, percentiles, statistics of local maxima (peaks), and linear prediction residuals (‘predictability’). More details are given in [57]. The motivation for using these features is that eating various types of food afflicts speech production in distinct ways and produces noises characteristic for the type of food; clearly, both speech production and noise effects are captured by the above-named features. Thus, they seem suitable for the 7-way task.

**Table 7:** ComParE acoustic feature set: 65 low-level descriptors (LLD).

**4 energy related LLD**	**Group**
Sum of auditory spectrum (loudness)	prosodic
Sum of RASTA-filtered auditory spectrum	prosodic
RMS Energy, Zero-Crossing Rate	prosodic
**55 spectral LLD**	**Group**
RASTA-filt. aud. spect. bands. 1–26 (0–8 kHz)	spectral
MFCC 1–14	cepstral
Spectral energy 250–650 Hz, 1 k–4 kHz	spectral
Spectral Roll-Off Pt. 0.25, 0.5, 0.75, 0.9	spectral
Spectral Flux, Centroid, Entropy, Slope	spectral
Psychoacoustic Sharpness, Harmonicity	spectral
Spectral Variance, Skewness, Kurtosis	spectral
**6 voicing related LLD**	**Group**
*F*_0_ (SHS & Viterbi smoothing)	prosodic
Prob. of voicing	voice qual.
log. HNR, Jitter (local & *δ*), Shimmer (local)	voice qual.

**Table 8:** ComParE acoustic feature set: Functionals applied to LLD contours (Table 7).

**Functionals applied to LLD / Δ LLD**	**Group**
quartiles 1–3, 3 inter-quartile ranges	percentiles
1% percentile (≈ min), 99% pctl. (≈ max)	percentiles
percentile range 1%–99%	percentiles
position of min / max, range (max—min)	temporal
arithmetic mean^1^, root quadratic mean	moments
contour centroid, flatness	temporal
standard deviation, skewness, kurtosis	moments
relative duration LLD is rising	temporal
rel. dur. LLD is above 25 / 50 / 75 / 90% range	temporal
rel. duration LLD has positive curvature	temporal
gain of linear prediction (LP), LP Coeff. 1–5	modulation
mean, max, min, std. dev. of segment length^2^	temporal
**Functionals applied to LLD only**	**Group**
mean value of peaks	peaks
mean value of peaks—arithmetic mean	peaks
mean / std.dev. of inter peak distances	peaks
amplitude mean of peaks, of minima	peaks
amplitude range of peaks	peaks
mean / std. dev. of rising / falling slopes	peaks
linear regression slope, offset, quadratic error	regression
quadratic regression a, b, offset, quadratic err.	regression
percentage of non-zero frames^3^	temporal

^1^: arithmetic mean of LLD / positive Δ LLD.

^2^: not applied to voicing related LLD except *F*_0_.

^3^: only applied to F0.

As classifier, we used SVMs, which are able to cope very well with a large size of the feature space. In a preliminary experiment, we obtained only limited performance gains by ‘tuning’ of the SVM parameters; thus, to favour easy and straight-forward reproducibility, we only report results using the default parameters, in particular using a complexity of 0.1 and a linear kernel, as we did for the ASR-related features set.

### Results and Discussion

Table 9 details the results on the binary task obtained by SVMs on ASR-related features. CER outperforms slightly but significantly (*p* < .05 according to a z-test) WER, probably due to its higher robustness (cf. above). Note that WER and CER results for spontaneous speech are not given because at the time of writing, we have not transcribed these utterances. Yet, it can also be seen that the best single feature is the real-time factor, which slightly outperforms other features that require the reference transcription. RTF also works robustly on spontaneous speech, which is not the case for the log-likelihood feature. Early fusion of the features, i. e., concatenating features before the classification step, shows that RTF and LL provide a complementary description of the eating condition, as they allow to improve the overall performance for both read and spontaneous speech production. For read speech, WER and CER also seem to entail information which is complementary to RTF and LL features; here, we obtained the best performance by fusing all ASR-related features.

**Table 9:** Binary classification of eating condition.

UAR [%]	Speech type		
Feature	Spontaneous	Read	All
WER	–	85.3	–
CER	–	90.7	–
RTF	86.9	91.8	91.8
LL	68.1	83.3	79.9
WER+CER	–	90.3	–
RTF+LL	**91.1**	93.9	**92.5**
ALL	–	**94.9**	–

Binary classification of eating condition (Food / No Food) using ASR-related features per type of speech (spontaneous and read as well as both): UAR using SVMs. WER and CER require knowledge of the reference text whereas RTF and LL do not require a priori knowledge. Chance level UAR: 50.0%. WER: word error rate, CER: character error rate, RTF: real-time factor, LL: log-likelihood.

Next, the UAR achieved on the binary and 7-way tasks by SVMs using the ComParE set of low-level acoustic features is shown in Table 10. Note that we evaluate separate classifiers for the binary and 7-way tasks rather than mapping the 7-way predictions to binary ones. Results obtained with the ASR-related features, as well as with the early fusion with the ComParE set are also shown in Table 10. The ComParE feature set provided a performance close to perfection for the binary classification task, and better than any performance attained by ASR-related features. In particular, the performance difference (ComParE set; all, read and spontaneous speech) to the best ASR feature (RTF) is statistically significant according to a z-test, *p* < .001. While this is partly expected due to the large size of the ComParE set, it is also notable because the ComParE features can be computed with an RTF ≪1 on a standard PC whereas ASR decoding showed much higher time complexity in our experiments—it remains to be investigated if decoding parameters can be tuned to speed up calculation without compromising the accuracy of eating condition classification. Although the ComParE feature set performed better than the ASR-related features, a slight improvement was obtained by the fusion of these two feature sets for the read speech condition.

**Table 10:** 2-way and 7-way classification of eating condition.

UAR [%]	Speech type			Chance
	Spontaneous	Read	All	
ASR-related				
2-way	91.1	94.9	92.5	50.0
7-way	28.1	31.2	30.0	14.3
ComParE				
2-way	**91.8**	98.0	**97.1**	50.0
7-way	**62.3**	65.6	**65.1**	14.3
ASR-related + ComParE				
2-way	89.6	**98.7**	96.9	50.0
7-way	57.2	**66.4**	65.0	14.3

2-way (Food / No Food) and 7-way classification of eating condition (6 types of food / No Food) using either ASR-related features, low-level acoustic features (ComParE set) or their combination, per type of speech (spontaneous and read as well as both): UAR using SVMs.

In the 7-way classification, robust results of 62.3% UAR on spontaneous speech—with slightly higher performance on read speech (65.6% UAR)—are obtained, which is more than four times higher than the chance level. Even if the performance obtained by the ASR-related features set (31.2% UAR) is two times lower than the one obtained with the ComParE feature set, an improvement was observed by the early fusion of these feature sets for the read speech condition. As might be expected, the overall performance obtained on spontaneous speech is always lower than for read speech, as it is easier for the latter condition to compare the acoustics of speech for different types of food under the same context of production.

In addition, the classifier confusions for the 7-way task and low-level acoustic features are shown in Table 11 as a matrix of the form *c*_*i*,*j*_ = Pr(predicted class *j* ∣ true class *i*). Thus, the diagonal of the matrix represents the class-wise recalls, and the average of those values represents the UAR. As would be expected, misclassifications occur mostly between food types that have similar consistence—Apple and Nectarine, Banana and Haribo Smurfs, and Biscuit and Crisps—and hence have a similar effect on pronunciation and sound in general. This notion of similarity motivates the introduction of a scale for eating conditions, as will be introduced next.

**Table 11:** Confusion matrix obtained by SVMs on the ComParE feature set in the 7-way classification of eating condition for both read and spontaneous speech production.

**[%]**	**Ap**	**Ne**	**Ba**	**Ha**	**Bi**	**Cr**	**No**
**Ap**	**55.1**	24.0	4.1	5.1	5.1	5.1	1.5
**Ne**	21.9	**44.9**	14.8	11.2	3.6	3.1	0.5
**Ba**	5.7	18.1	**48.6**	22.4	1.9	0.5	2.9
**Ha**	6.9	11.1	7.9	**69.8**	0.5	2.1	1.6
**Bi**	7.9	6.4	1.0	1.0	**72.4**	10.8	0.5
**Cr**	6.7	3.8	1.0	3.3	12.4	**72.4**	0.5
**No**	0.5	1.4	3.8	1.0	0.5	0.5	**92.4**

## A Regression Approach

In CP, there is a long tradition to go from multi-way classification problems to dimensional modelling [58] and recognition through regression, e. g., emotion recognition in the arousal-valence space [41, 59, 60]. Here, we follow a data-based approach to compute possible one-to-one correspondences between a set of *C* discrete classes and points in *K*-D space. The premise is that similar classes should be represented by points that lie close together. This can be realised by applying Non-metric Multi-Dimensional Scaling (NMDS) to a symmetric matrix $D\in R_{+}^{C\times C}$ of class dissimilarities, as performed in [19]. For instance, assuming that similar classes are easily confused, we can obtain such a matrix from the class confusion matrix **C** ∈ [0, 1]^7×7^ (cf. Table 11) as

$$D=\left(d_{i,j}\right):=1-\frac{1}{2}\left(C+C^{⊺}\right).$$ (1)

Then, Sammon’s method [61] is used to find a representation of the classes as vectors $m_{i}\in R^{K}$, *i* = 1, …, *C*, such that the pair-wise distances ${\hat{d}}_{i,j}=|m_{i}-m_{j}{|}_{2}$ are correlated with the original dissimilarities *d*_*i*,*j*_ (cf. (Eq 1)).

**Fig 2:**
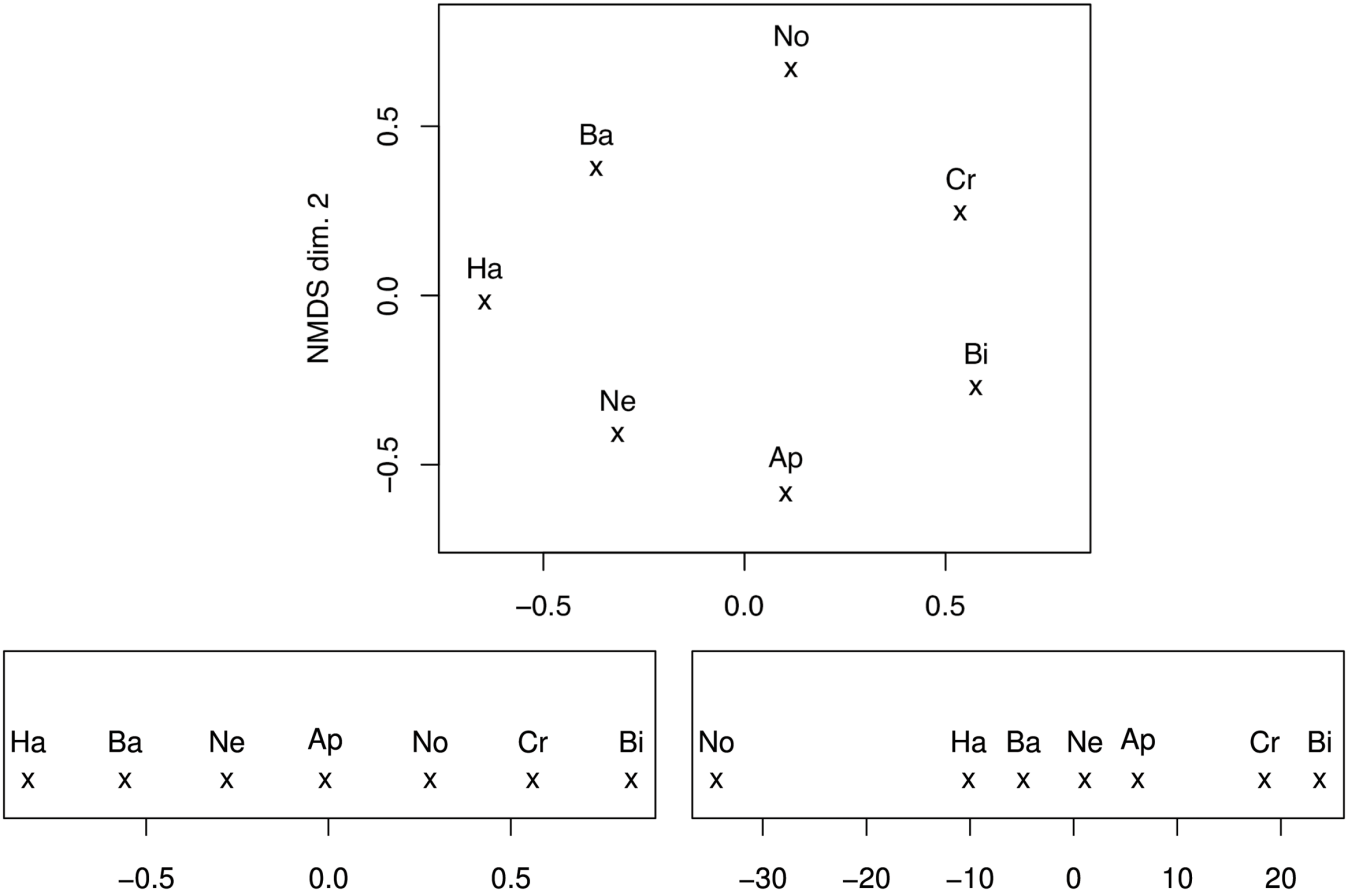
Solutions of non-metric dimensional scaling applied to class confusions (2-D (top), 1-D (bottom left)) or Euclidean class center distances (1-D (bottom right)) in the 7-way task, ComParE low-level acoustic features.

For *K* = 2, the stress value (normalised sum of squared errors) after termination of the algorithm is 0.069 and the *R*^2^ of $\hat{D}$ and **D** is 0.46; the resulting configuration is shown in Fig 2 (top). For *K* = 1, we obtained a solution with stress = 0.215 and *R*^2^ = 0.28, which is shown in Fig 2 (bottom left). According to the ordering of classes along the NMDS axis, this dimension can be interpreted as the ‘crispiness’ of the food (ranging from Haribo Smurfs to Biscuits), with a ‘neutral’ point in the middle (No Food). It is obvious that the 2-D solution (Fig 2 (top)) has a circular shape with a first dimension that is highly similar to the 1-D solution, yet a hardly interpretable second dimension; thus, we opt for the 1-D representation for the following analyses.

We can alternatively define *d*_*i*,*j*_ as the Euclidean distance of the class centres *i* and *j* in the ComParE acoustic feature space. In fact, this yields a 1-D solution with much lower stress and higher *R*^2^ (stress = 0.073, *R*^2^ = .85), which exhibits exactly the same ordering of the six food types along the axis, yet with a distinct separation of ‘crispy’ foods (Crisps, Biscuit), and No Food being an outlier. This solution is shown in Fig 2 (bottom right).

**Fig 3:**
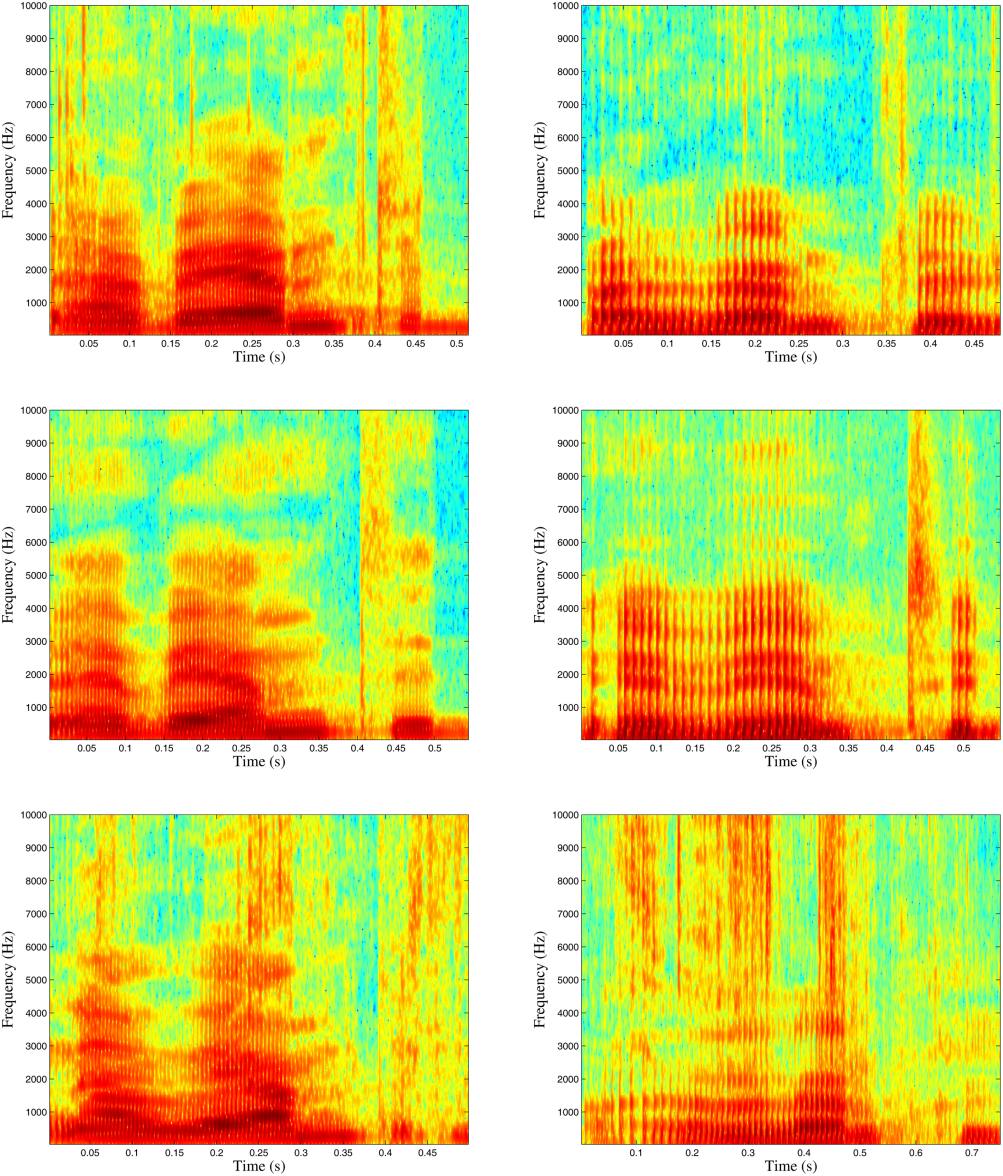
Degree of high-frequency noise of the words ‘warmed up’ caused by eating. Subjects (left: female, right: male) while recording an utterance eating a banana (top), without eating a sort of food (middle), and eating crisps (bottom).

One possible explanation for the latter solution is the degree of high-frequency noise caused by eating food, which is expected to be absent in the case of No Food and strongly present in the case of ‘crispy’ food. Fig 3 shows the frequency spectrum of the word ‘warmed up’ from a female (left) and a male subject (right) while recording an utterance first while eating a banana (top), then without eating while speaking (middle), and finally eating crisps (bottom). Compared to No Food, formants present considerably less intensity for Banana, which might be due to the obtrusion of the air flow in the mouth cavity resulting from the presence of the piece of banana. Crisps generate furthermore a high level of noise in the high-frequencies, which might be due to the sound generated when crushing the piece of crispy food.

In particular, we found functionals of the highest auditory frequency bands to be the most predictive features (in terms of *R*^2^). For instance, the flatness of the delta coefficients of the 23rd frequency band, ranging from 5.28 kHz to 6.49 kHz, accounts for 28.5% of the variance of the ‘crispiness’ dimension (t = 24.2, *p* < .001). We argue that this feature indicates a prevalence of rapid energy changes in this frequency range (for crispy food), as opposed to slow changes (for less crispy food or no food).

Conversely, the solution Fig 2 (bottom right) with No Food in the middle might simply be due to this class being robustly discriminated from the others, thus being equally dissimilar from all other classes. Furthermore, the low *R*^2^ value for this solution might indicate that it is less meaningful. However, in our data we still found some acoustic features that are correlated with the ordering of classes in this solution, such as residual energies of linear prediction of cepstra—e.g., the residual of the prediction of the third delta MFCC accounts for 23.0% of the variance (t = -13.0, *p* < .001). Apparently, jelly-like foods cause ‘predictable’ phonetic content due to a smoothing effect of impeded speech production, while crispy foods cause ‘unpredictable’ changes, and ‘clean’ speech is unaffected by either of these phenomena.

We can now move on to automatic regression experiments using the numeric labels derived from the 1-D NMDS solutions, i. e., by exchanging the class labels with the abscissae displayed in Fig 2 (bottom left or bottom right). To this end, we train and evaluate linear support vector regression (SVR) models in LOSO-CV, using the 100 most relevant features per fold selected by *R*^2^ with the label (on the training set of each fold). The implementation in Weka’s SMOreg class is used, with a complexity of 0.1 as for SVM. The evaluation metrics are *R*^2^ and relative absolute error (RAE), which is the mean absolute error normalised by the chance level mean absolute error (by always predicting the mean label). Consequently, the chance level RAE is 100% while the corresponding *R*^2^ is always zero. A more meaningful baseline for regression is the *R*^2^ of the best single feature and the labels (cf. above on the most predictive feature types). From Table 12, one can see that regression generally delivers robust results, outperforming the single feature baseline by more than 20% absolute in *R*^2^. Furthermore, since regression on the class-center-distance-based labels works better (11% absolute gain in *R*^2^), while both labels are based on the same acoustic information, there is significant evidence that the distance-based ones are a more meaningful dimensional representation.

**Table 12:** Regression-based recognition of eating condition.

[%]	SVR/100 feat.		1-best feat.
Label	*R* ^2^	RAE	*R* ^2^
NMDS 1-D (Conf)	45.5	69.7	23.0
NMDS 1-D (Dist)	**56.2**	66.8	31.9

Regression-based recognition of eating condition: Determination coefficient (*R*^2^) and relative absolute error (RAE) obtained by SVR on the ComParE feature set, and best single feature baseline. Numeric labels obtained from 1-D NMDS solutions (‘Conf’usions or ‘Dist’ance of class centers).

## iHEARu-EAT as part of the INTERSPEECH 2015 Computational Paralinguistics Challenge

As the 7-way classification task was featured as a sub-challenge in the INTERSPEECH 2015 Computational Paralinguistics Challenge (ComParE) [46], which is a comparative evaluation of signal processing and machine learning approaches run under the exact equal conditions for all participants, we can expect further insight into this classification problem.

For the challenge, the iHEARu-EAT database was partitioned into a training (20 speakers) and a testing (10 speakers) set. The results described in the challenge and those presented in this article are thus not directly comparable, as we used in this paper a LOSO evaluation scheme. Since the performance on the 2-way classification task was almost perfect, we decided to use the 7-way classification task for the challenge, which provides a much larger room for improvement compared to the binary classification task. The baseline system was build with the same acoustic features set (ComParE 2013) and machine learning algorithm (SVM) as used in this study. Optimisation on the complexity of the SVM was, however, performed for the Challenge, by carrying out cross-validation on the training set. The obtained UAR baseline performance of the proposed system was 65.9%.

All in all, 14 sites participated in this sub-challenge, from which seven papers were accepted by the technical program committee. A large variety of techniques, ranging from feature selection techniques to (deep) neural networks, were proposed by the participants of the challenge to solve the 7-way classification task. The use of Softmax and single hidden-layer neural networks using Rectified Linear Units activation function was, for example, proposed in [62]. SVM based hierarchical classification was addressed in [63], whereas a wrapper-based feature selection approach, which combines a feature subset evaluation and a feature space search, was addressed in [64]. Further, a rather unusual approach was followed in [65]: Instead of suppressing the influence of noise to enhance the intelligibility of a spoken message, the authors emphasised the noisy parts of the spectrum to improve the recognition of food classes. [66] built upon a deep learning language identification system, with the main idea to train a local convolutional neural network classifier. A system, which incorporates i-vectors and functionals for segmental features, non-linear time series features, speech rhythm, and ASR decoding based features was proposed in [67].

Most of the contributions yield a performance above the baseline and up to 83.1% for the winning team [68]. This best performing team has shown that the task is not only a question of ‘brute-forcing’ but rather of compensating speaker variabilities to foster differences generated by the eating conditions. The variability compensation issue by proposing a novel method composed of Fisher vector encoding of LLDs, speaker z-normalisation applied after speaker clustering, non-linear normalisation of features, and classification based on Kernel Extreme Learning Machines and Partial Least Squares regression made this team the winning team.

## Conclusions and Outlook

We have introduced the iHEARu-EAT database and performed experiments that demonstrate the feasibility of automatic classification of eating conditions under speaking. There exists previous work on acoustic-based recognition of eating, albeit mostly with special hardware requirements in terms of the sensor. Further, usually eating is not considered under speech, making our database a first of its kind.

First, the impact of training with speech data while eating and without eating on the 7-class recognition task has been shown. It has been confirmed that training on a single kind of food performs better in matched testing conditions than training on all types of food. So, ASR for speech under eating deteriorates when trained with clear speech, and can benefit from modelling different types of eating.

Further, while single, intelligibility-related ASR features are sufficient for binary classification, the importance of low-level acoustic features for finer-grained discrimination has been confirmed, and the complementarity of both have been shown for read speech. Acoustic features are also consistent with a dimensional modelling of the eating condition.

Limitations of this study can be found in the exemplary but necessarily sparse choice of food types. For specific applications, of course, other types of food had to be selected. Further, we did not exert full experimental control over all parameters that might be relevant, which pertains the structure and texture of the food chosen [69–71], as well as speaker characteristics such as the vocal tract length and personality ‘background’ which might cause different ways of chewing and accordingly [72] slightly different acoustic characteristics. We do not know yet whether and if such differences might influence or even impede modelling and classification performance. Note, however, that we tried to take into account such random factors by employing a non-metric procedure for dimensional modelling and interpretation. Further, there are, of course, other questions here as well regarding the physiology of speech and eating which are not explored so far.

In the future, we will investigate deeper into the meaning of single acoustic features, and use some segmentation into sub-utterance units to segregate the classification of acoustic events (such as chewing) and speech ‘impairments’ due to eating. Furthermore, as we found that different test subjects exhibit distinct behaviour in speaking while eating, speaker adaptation will be considered. The self-reports provided on the likability and difficulty of eating will also be considered as new prediction tasks. As additional annotations to the corpus, we plan to manually transcribe the spontaneous speech utterances to enable ASR performance studies. Finally, we will also exploit the visual channel to obtain complementary information for multimodal fusion.

## References

[1] Schuller B, Batliner A. Computational Paralinguistics—Emotion, Affect, and Personality in Speech and Language Processing. Chichester, UK: Wiley; 2014.

[2] Schuller B, Zhang Y, Eyben F, Weninger F. Intelligent systems’ Holistic Evolving Analysis of Real-life Universal speaker characteristics. In: Schuller B, Buitelaar P, Devillers L, Pelachaud C, Declerck T, Batliner A, et al., editors. Proceedings of the 5th International Workshop on Emotion Social Signals, Sentiment & Linked Open Data (ES^3^LOD 2014), satellite of the 9th Language Resources and Evaluation Conference (LREC 2014). Reykjavik, Iceland: ELRA; 2014. p. 14–20.

[3] Womack BD, Hansen JHL. N-channel hidden Markov models for combined stressed speech classification and recognition. IEEE Transactions on Speech and Audio Processing. 1999;7(6):668–677. 10.1109/89.799692

[4] Wu T, Yang Y, Wu Z. Improving Speaker Recognition by Training on Emotion-Added Models In: Tao J, Tan T, Picard RW, editors. Proc. of ACII 2005. Berlin, Heidelberg: Springer; 2005 p. 382–389.

[5] van Doremalen J, Cucchiarini C, Strik H. Optimizing Automatic Speech Recognition for Low-Proficient Non-Native Speakers. EURASIP Journal on Audio, Speech, and Music Processing. 2010:1–13. 10.1155/2010/973954

[6] Neumeyer L, Franco H, Digalakis V, Weintraub M. Automatic scoring of pronunciation quality. Speech Communication. 2000;30:83–93. 10.1016/S0167-6393(99)00046-1

[7] Hönig F, Batliner A, Nöth E. Automatic Assessment of Non-Native Prosody—Annotation, Modelling and Evaluation. In: Proc. of the International Symposium on Automatic Detection of Errors in Pronunciation Training (isadept). Stockholm; 2012. p. 21–30.

[8] Scipioni M, Gerosa M, Giuliani D, Nöth E, Maier A. Intelligibility Assessment in Children with Cleft Lip and Palate in Italian and German. In: Proc. of INTERSPEECH. Brighton; 2009. p. 967–970.

[9] Maier A, Haderlein T, Eysholdt U, Rosanowski F, Batliner A, Schuster M, et al PEAKS—A system for the automatic evaluation of voice and speech disorders. Speech Communication. 2009;51:425–437. 10.1016/j.specom.2009.01.004

[10] Middag C, Bocklet T, Martens JP, Nöth E. Combining phonological and acoustic ASR-free features for pathological speech intelligibility assessment. In: Proc. of INTERSPEECH. Florence, Italy; 2011. p. 3005–3008.

[11] Ringeval F, Demouy J, Szaszák G, Chetouani M, Robel L, Xavier J, et al Automatic Intonation Recognition for Prosodic Assessment of Language Impaired Children. IEEE Transactions on Audio, Speech & Language Processing. 2011;19(5):1328–1342. 10.1109/TASL.2010.2090147

[12] Schuller B, Steidl S, Batliner A, Schiel F, Krajewski J, Weninger F, et al Medium-Term Speaker States—A Review on Intoxication, Sleepiness and the First Challenge. Computer Speech and Language, Special Issue on Broadening the View on Speaker Analysis. 2014;28(2):346–374.

[13] Flege JE, Fletcher SG, Homiedan A. Compensating for a bite block in /s/ and /t/ production: Palatographic, acosutic, and perceptual data. J Acoust Soc Am. 1988;83:212–228. 10.1121/1.396424 3343441

[14] Hiiemae KM, Palmer JB, Medicis SW, Hegener J, Jackson BS, Lieberman DE. Hyoid and tongue surface movements in speaking and eating. Archives of Oral Biology. 2002;47:11–27. 10.1016/S0003-9969(01)00092-9 11743928

[15] Vennemann T. Was isst und was soll die Phanologie? In: Nordica et Mystica. Festschrift für Kurt Schier. München: Institut für Nordische Philologie; 1979 p. 64–83.

[16] Mayer C, Gick B. Talking while Chewing: Speaker Response to Natural Perturbation of Speech. Phonetica. 2012;69(3):109–123. 10.1159/000336117 23258462

[17] Dacremont C. Spectral composition of eating sounds generated by crispy, crunchy and crackly foods. Journal of texture studies. 1995;26(1):27–44. 10.1111/j.1745-4603.1995.tb00782.x

[18] Vickers ZM. Pleasantness of Food Sounds. Journal of Food Science. 1983;48(3):783–786. 10.1111/j.1365-2621.1983.tb14898.x

[19] Vickers ZM, Wasserman SS. Sensory qualities of food sounds based on individual perceptions. Journal of Texture Studies. 1983;10(4):319–332. 10.1111/j.1745-4603.1980.tb00863.x

[20] Vickers ZM. Food sounds: how much information do they contain? Journal of Food Science. 1980;45(6):1494–1496.

[21] David GC, Garcia AC, Rawls AW, Chand D. Listening to what is said—transcribing what is heard: the impact of speech recognition technology (SRT) on the practice of medical transcription (MT). Sociology of Health & Illness. 2009;31(6):924–938. 10.1111/j.1467-9566.2009.01186.x 19843274

[22] Steidl S, Batliner A, Seppi D, Schuller B. On the Impact of Children’s Emotional Speech on Acoustic and Language Models. EURASIP Journal on Audio, Speech, and Music Processing, Special Issue on Atypical Speech. 2010(Article ID 783954):1–14. 10.1155/2010/783954

[23] Geiger JT, Zhang B, Schuller B, Rigoll G. On the Influence of Alcohol Intoxication on Speaker Recognition. In: Proceedings AES 53rd International Conference Semantic Audio. AES. London, UK: Audio Engineering Society; 2014. p. 1–7.

[24] Puri M, Zhu Z, Yu Q, Divakaran A, Sawhney H. Recognition and Volume Estimation of Food Intake using a Mobile Device. In: WACV 2009: Proceedings of the Workshop on Applications of Computer Vision. IEEE; 2009. p. 1–8.

[25] Zhang S, MH AJ, Xiao W, Tham CK. Detection of Activities by Wireless Sensors for Daily Life Surveillance: Eating and Drinking. Sensors. 2009;9:1499–1517. 10.3390/s90301499 22573968 PMC3345819

[26] Alshurafa N, Kalantarian H, Pourhomayoun M, Sarin S, Liu JJ, Sarrafzadeh M. Non-Invasive Monitoring of Eating Behavior using Spectrogram Analysis in a Wearable Necklace. In: HIC: Proceedings of the Healthcare Innovation Conference. IEEE; 2014. p. 71–74.

[27] Sazonov ES, Fontana JM. A Sensor System for Automatic Detection of Food Intake Through Non-Invasive Monitoring of Chewing. IEEE Sensors Journal. 2012;12(5):1340–1348. 10.1109/JSEN.2011.2172411 22675270 PMC3366471

[28] Makeyev O, Lopez-Meyer P, Schuckers S, Besio W, Sazonov E. Automatic food intake detection based on swallowing sounds. Biomedical Signal Processing and Control. 2012;7(6):649–656. 10.1016/j.bspc.2012.03.005 23125873 PMC3483798

[29] Passler S, Fischer W, Kraljevski I. Adaptation of Models for Food Intake Sound Recognition Using Maximum a Posteriori Estimation Algorithm. In: Wearable and Implantable Body Sensor Networks (BSN), 2012 Ninth International Conference on. London, UK: IEEE; 2012. p. 148–153.

[30] Amft O. Automatic dietary monitoring using on-body sensors: Detection of eating and drinking behaviour in healthy individuals. ETH Zurich; 2008.

[31] Sazonov ES, Schuckers S, Neuman MR. Automatic Detection of Swallowing Events by Acoustical Means for Applications of Monitoring of Ingestive Behavior. IEEE Transactions on Biomedical Engineering. 2010;57(3):626–633. 10.1109/TBME.2009.2033037 19789095 PMC2886152

[32] Päßler S, Wolff M, Fischer WJ. Food intake monitoring: an acoustical approach to automated food intake activity detection and classification of consumed food. Physiological Measurement. 2012;33:1073–1093. 10.1088/0967-3334/33/6/1073 22621915

[33] Pfeiffer RF, Wszolek ZK, Ebadi M. Parkinson’s Disease. vol. 2 CRC Press; 2012.

[34] Karkos PD. Current evaluation of the dysphagic patient. Hippokratia. 2009;13(3):141–146. 19918301 PMC2765291

[35] Bernstein M, Luggen A. Nutrition for the older adult. Jones & Bartlett Learning; 2010.

[36] Yatani K, Truong KN. BodyScope: A Wearable Acoustic Sensor for Activity Recognition. In: UbiComp’12: Proceedings of the 2012 ACM Conference on Ubiquitous Computing. ACM; 2012. p. 341–350.

[37] Rahman T, Adams AT, Zhang M, Cherry E, Zhou B, Peng H, et al. BodyBeat: A Mobile System for Sensing Non-Speech Body Sounds. In: MobiSys’14: Proceedings of the 12th annual international conference on Mobile systems, applications, and services. ACM; 2014. p. 2–13.

[38] Amft O, Stäger M, Lukowicz P, Tröster G. Analysis of Chewing Sounds for Dietary Monitoring. In: UbiComp 2005: Proceedings of the 7th International Conference on Ubiquitous Computing. Tokyo, Japan; 2005. p. 56–72.

[39] Vacher M, Fleury A, Portet F, Serignat JF, Noury N. Complete Sound and Speech Recognition System for Health Smart Homes: Application to the Recognition of Activities of Daily Living New Developments in Biomedical Engineering. 2010; p. 645–673.

[40] Schuller B, Dunwell I, Weninger F, Paletta L. Serious Gaming for Behavior Change—The State of Play. IEEE Pervasive Computing Magazine, Special Issue on Understanding and Changing Behavior. 2013;12(3):48–55. 10.1109/MPRV.2013.54

[41] Ringeval F, Eyben F, Kroupi E, Yuce A, Thiran JP, Ebrahimi T, et al Prediction of asynchronous dimensional emotion ratings from audiovisual and physiological data. Pattern Recognition Letters. 2015;66:22–30. 10.1016/j.patrec.2014.11.007

[42] Hymes D. Introduction: Toward Ethnographies of Communication. American Anthropologist. 1964;66:1–34. 10.1525/aa.1964.66.suppl_3.02a00010

[43] Hymes D. The Ethnography of Speaking In: Gladwin T, Sturtevant WC, editors. Anthropology and Human Behavior. Anthropological Society of Washington; 1962 p. 13–53.

[44] Bendix R. Reden und Essen: Kommunikationsethnographische Ansätze zur Nahrungsethnologie. Österreichische Zeitschrift für Volkskunde. 2004;107:211–238.

[45] Elias N. Über den Prozeß der Zivilisation. Soziogenetische und psychogenetische Untersuchungen Band 1: Wandlungen des Verhaltens in den weltlichen Oberschichten des Abendlandes / Band 2: Wandlungen der Gesellschaft: Entwurf zu einer Theorie der Zivilisation. Basel: Verlag Haus zum Falken; 1939.

[46] Schuller B, Steidl S, Batliner A, Hantke S, Hönig F, Orozco-Arroyave JR, et al. The INTERSPEECH 2015 Computational Paralinguistics Challenge: Degree of Nativeness, Parkinson’s & Eating Condition. In: Proc. of INTERSPEECH. Dresden, Germany: ISCA; 2015. p. 478–482.

[47] Haderlein T, Moers C, Möbius B, Rosanowski F, Nöth E. Intelligibility Rating with Automatic Speech Recognition, Prosodic, and Cepstral Evaluation. In: Proceedings of Text, Speech and Dialogue (TSD). vol. 6836 of Lecture Notes in Artificial Intelligence. Berlin, Heidelberg: Springer; 2011. p. 195–202.

[48] Povey D, Ghoshal A, Boulianne G, Burget L, Glembek O, Goel N, et al. The Kaldi speech recognition toolkit. In: IEEE 2011 workshop on automatic speech recognition and understanding. EPFL-CONF-192584. IEEE Signal Processing Society; 2011. four pages.

[49] Novak JR, Minematsu N, Hirose K. Failure Transitions for Joint N-gram Models and G2P Conversion. In: INTERSPEECH. Lyon, France; 2013. p. 1821–1825.

[50] Kneser R, Ney H. Improved backing-off for M-gram language modeling. In: ICASSP. vol. 1. Detroit, Michigan, USA; 1995. p. 181–184.

[51] Stolcke A. SRILM—an extensible language modeling toolkit. In: ICSLP. vol. 2. Denver, Colorado, USA; 2002. p. 901–904.

[52] McGraw KO, Wong S. A common language effect size statistic. Psychological Bulletin. 1992;111(2):361–365. 10.1037/0033-2909.111.2.361

[53] Schuller B. The Computational Paralinguistics Challenge. IEEE Signal Processing Magazine. 2012;29(4):97–101. 10.1109/MSP.2012.2192211

[54] Weninger F, Schuller B, Eyben F, Wöllmer M, Rigoll G. A Broadcast News Corpus for Evaluation and Tuning of German LVCSR Systems. CoRR. 2014;abs/1412.4616.

[55] Schuller B, Steidl S, Batliner A, Vinciarelli A, Scherer K, Ringeval F, et al. The INTERSPEECH 2013 Computational Paralinguistics Challenge: Social Signals, Conflict, Emotion, Autism. In: Proc. of INTERSPEECH. Lyon, France: ISCA; 2013. p. 148–152.

[56] Eyben F, Weninger F, Groß F, Schuller B. Recent Developments in openSMILE, the Munich Open-Source Multimedia Feature Extractor. In: Proc. of ACM MM. Barcelona, Spain: ACM; 2013. p. 835–838.

[57] Weninger F, Eyben F, Schuller B, Mortillaro M, Scherer KR. On the Acoustics of Emotion in Audio: What Speech, Music and Sound have in Common. Frontiers in Emotion Science. 2013;4(292):1–12. 10.3389/fpsyg.2013.00292 PMC3664314 23750144

[58] Batliner A, Steidl S, Hacker C, Nöth E. Private emotions vs. social interaction—a data-driven approach towards analysing emotions in speech. User Modeling and User-Adapted Interaction. 2008;18:175–206. 10.1007/s11257-007-9039-4

[59] Wöllmer M, Eyben F, Reiter S, Schuller B, Cox C, Douglas-Cowie E, et al. Abandoning Emotion Classes—Towards Continuous Emotion Recognition with Modelling of Long-Range Dependencies. In: Proc. of INTERSPEECH. Brisbane, Australia: ISCA; 2008. p. 597–600.

[60] Yang YH, Lin YC, Su YF, Chen HH. A Regression Approach to Music Emotion Recognition. IEEE Transactions on Audio, Speech and Language Processing. 2008;16(2):448–457. 10.1109/TASL.2007.911513

[61] Sammon JW. A Nonlinear Mapping for Data Structure Analysis. IEEE Transactions on Computers. 1969;C-18(5):401–409. 10.1109/T-C.1969.222678

[62] Pellegrini T. Comparing SVM, Softmax, and shallow neural networks for eating condition classification. In: Proc. of INTERSPEECH. Dresden, Germany: ISCA; 2015. p. 899–903.

[63] Prasad A, Ghosh PK. Automatic Classification of Eating Conditions from Speech Using Acoustic Feature Selection and a Set of Hierarchical Support Vector Machine Classifiers. In: Proc. of INTERSPEECH. ISCA. Dresden, Germany: ISCA; 2015. p. 884–888.

[64] Pir D, Brown T. Acoustic Group Feature Selection Using Wrapper Method for Automatic Eating Condition Recognition. In: Proc. of INTERSPEECH. Dresden, Germany: ISCA; 2015. p. 894–898.

[65] Wagner J, Seiderer A, Lingenfelser F, André E. Combining Hierarchical Classification with Frequency Weighting for the Recognition of Eating Conditions. In: Proc. of INTERSPEECH. Dresden, Germany: ISCA; 2015. p. 889–893.

[66] Milde B, Biemann C. Using Representation Learning and Out-of-domain Data for a Paralinguistic Speech Task. In: Proc. of INTERSPEECH. Dresden, Germany: ISCA; 2015. p. 904–908.

[67] Kim J, Nasir M, Gupta R, Segbroeck M, Bone D, Black M, et al. Automatic estimation of Parkinson’s disease severity from diverse speech tasks. In: Proc. of INTERSPEECH. Dresden, Germany: ISCA; 2015. p. 914–918.

[68] Kaya H, Karpov AA, Salah AA. Fisher Vectors with Cascaded Normalization for Paralinguistic Analysis. In: Proc. of INTERSPEECH. Dresden, Germany: ISCA; 2015. p. 909–913.

[69] Fillion L, Kilcast D. Consumer perception of crispness and crunchiness in fruits and vegetables. Food Quality and Preference. 2002;13(1):23–29. 10.1016/S0950-3293(01)00053-2

[70] Jowitt R. The terminology of food texture. Journal of Texture Studies. 1974;5(3):351–358. 10.1111/j.1745-4603.1974.tb01441.x

[71] Chew C, Lucas P, Tay D, Keng S, Ow R. The effect of food texture on the replication of jaw movements in mastication. Journal of dentistry. 1988;16(5):210–214. 10.1016/0300-5712(88)90072-3 3216059

[72] Sazonov E, Schuckers S, Lopez-Meyer P, Makeyev O, Sazonova N, Melanson EL, et al Non-invasive monitoring of chewing and swallowing for objective quantification of ingestive behavior. Physiological Measurement. 2008;29(5):525 10.1088/0967-3334/29/5/001 18427161 PMC2582220

